# Pooled Testing for Expanding COVID-19 Mass Surveillance

**DOI:** 10.1017/dmp.2020.246

**Published:** 2020-07-14

**Authors:** Angela Felicia Sunjaya, Anthony Paulo Sunjaya

**Affiliations:** Faculty of Medicine, Tarumanagara University, Jakarta, Indonesia; One Med Research Institute, Jakarta, Indonesia; Respiratory Division, The George Institute for Global Health, UNSW Sydney, Australia

**Keywords:** COVID-19, mass surveillance, pooled testing, population screening

## Abstract

Diagnostic testing to identify patients infected with severe acute respiratory syndrome coronavirus 2 (SARS-CoV-2) plays a key role to control the coronavirus disease (COVID-19) pandemic. While several countries have implemented the use of diagnostic testing in a massive scale as a cornerstone for infection control and surveillance, other countries affected by the pandemic are hampered by its limited testing capacity. Pooled testing was first introduced in the 1940s and is now used for screening in blood banks. Testing is done by pooling multiple individual samples together. Only in the case of a positive pool test would individual samples of the pool be tested, thus substantially reducing the number of tests needed. Several studies regarding their use for SARS CoV-2 have been done in the United States, Israel, and Germany. Studies have shown that an individual positive sample can still be detected in pools of up to 32 samples, and possibly even 64 samples, provided that additional polymerase chain reaction (PCR) amplification cycles are conducted with a sensitivity of 96%. Simulation studies to determine optimal pool size and pooling techniques have also been conducted. Based on these studies, pooled testing is shown to be able to detect positive samples with sufficient accuracy and can easily be used with existing equipment and personnel for population-wide screening.

Diagnostic testing to identify patients infected with severe acute respiratory syndrome coronavirus 2 (SARS-CoV-2) plays a key role to control the coronavirus disease (COVID-19) pandemic. While several countries have implemented the use of diagnostic testing in a massive scale as a cornerstone for infection control and surveillance, other countries affected by the pandemic are hampered by its limited testing capacity.^1^ Diagnostic identification of all individuals infected by SARS-CoV-2, including screening asymptomatic persons in the incubation phase, is crucial to limit viral spread. Innovations supporting population-based mass testing are required.

Pooled testing was first introduced in the 1940s and is now used for screening in blood banks.^2^ Testing is done by pooling multiple individual samples together, which are later tested with usual reverse transcriptase polymerase chain reaction (RT-PCR) systems. Only in the case of a positive pool test would individual samples of the pool be tested, substantially reducing the number of tests needed.^3^ It requires no additional training, equipment, or materials with several studies for SARS-CoV-2 already done in the United States,^2,4^ Israel,^5^ and Germany.^6^

Previous studies show that individual positive samples of SARS-CoV-2 can still be detected in pools of up to 32 samples, and possibly even 64 samples, provided that additional PCR amplification cycles are done. Sensitivity for a pool size of 16 samples was 96% with an estimated false negative of 10%. Pooled testing could also potentially be applied prior to ribonucleic acid (RNA) extraction, thus saving invaluable time and resources.^5^ Similar results were obtained by Abdalhamid et al., which were an increase in testing capacity by at least 69% when prevalence rates are 10% or less.^4^

Hogan et al. tested 2888 individual nasopharyngeal or bronchoalveolar lavage samples grouped in pools of 9 to 10. Two positive samples were identified for a positivity rate of 0.07% and only 1 false positive reading was observed.^4^ In Germany, pooled testing of 1191 samples in pool sizes between 4 and 30 samples resulted in only 267 tests required to detect 23 positive individuals (positive rate of 1.93%) with all positive samples easily identified.^6^

In Germany, 2 pooling techniques were compared, a “routine high throughput” approach where random samples are pooled together for testing or a “door to door” approach where groups of similar people (ie, families, neighbors, etc.) are pooled together for testing. While both approaches save substantial resources, the “door to door” approach was found to carry more benefit, reducing tests by 56% to 93%, whereas the “routine high throughput” resulted in 24% to 86% fewer tests. In low to moderate infection levels, even a pool size of 5 would reduce the number of tests needed by 5-fold (78%). In countries with infection levels over 20%, a pool size of 10 would still result in a considerable reduction in the number of tests required (up to 50%).^1^

These studies showed that pooled testing is able to detect positive samples with sufficient accuracy. It is best used for population-wide screening, contact tracing, and the monitoring of essential workers and asymptomatic individuals with unidentified risk, such as in airports, versus being much less effective if used in settings with high clinical suspicion, such as patients showing symptoms.

While concerns exist that low positive samples such as those found in convalescent patients could escape detection with increasing pool size, additional amplification cycles could be employed to allow better detection of larger pools.^2,5^ Nevertheless, pooled testing shows great potential in increasing testing capacity with existing resources with minimal loss of accuracy.
